# Pentoxifylline decreases glycemia levels and TNF-alpha, iNOS and COX-2 expressions in diabetic rat pancreas

**DOI:** 10.1186/2193-1801-3-283

**Published:** 2014-06-05

**Authors:** Francisca Adilfa O Garcia, Sofia F Pinto, Andrezza F Cavalcante, Lívia T Lucetti, Silvana MS Menezes, Cícero Francisco B Felipe, Ana Paula NN Alves, Gerly Anne C Brito, Gilberto S Cerqueira, Glauce SB Viana

**Affiliations:** Faculty of Medicine of the Federal University of Ceará, Fortaleza, Brazil; Faculty of Medicine Estácio of Juazeiro do Norte, Juazeiro do Norte, Brazil

**Keywords:** Pentoxifylline, Diabetes mellitus, Inflammation, Oxidative stress, Cytokines

## Abstract

Pentoxifylline (PTX), a methyl xanthine derivative, is a phosphodiesterase inhibitor with anti-inflammatory and renoprotective effects in diabetic patients, among other properties. We studied PTX actions and mechanisms in reducing blood biochemical parameters, in diabetic rats. For diabetes induction, alloxan was intravenously administered to male Wistar rats. One group was left untreated and the other ones treated with PTX (25, 50 and 100 mg/kg), glibenclamide or metformin, as references. Forty-eight hours later and after 1-week to 3-month treatments, blood was collected for determination of glycemia, triglycerides, cholesterol, transaminases, fructosamine and glycated hemoglobin. Afterwards, the animals were euthanized and pancreas, liver and kidney processed for histological analyses and immunohistochemistry assays for TNF-alpha, iNOS and COX-2. The results showed that PTX decreased glycemia and also triglyceride levels, starting 1 week after treatments, as compared to the same group before treatments. Glycemia values were brought towards normality, after 1-month treatment. PTX hypoglycemic effects were potentiated by glibenclamide but not by metformin. It also decreased fructosamine and glycated hemoglobin. Some histological and immunohistochemical alterations for TNF-alpha, iNOS and COX-2 in the diabetic pancreas were also reversed by PTX. We conclude that PTX acts similarly to glibenclamide, and its hypoglycemic actions are, partly, a consequence of ATP-sensitive K^+^ channels inhibition. In addition, by its anti-inflammatory and antioxidant properties, PTX may be a therapeutic alternative for the treatment of diabetes and its complications.

## Introduction

Diabetes mellitus is a serious worldwide health problem associated with acute and chronic complications. In type 2 diabetes, either the body does not produce enough insulin or the cells ignore the insulin necessary for the use of glucose as an energy supply. Insulin takes the sugar from the blood into the cells. Diabetes complications occur when glucose builds up in the blood instead of going into the cells. Diabetes mellitus has been proposed (Hooper & Hooper 2009) to be a vicious cycle of metabolically induced inflammation, impaired insulin responsiveness and loss of homeostatic signaling.

In the past years, researches have established a clear relationship between chronic low-grade inflammation, obesity and insulin resistance. Adipocytes and macrophages secrete inflammatory cytokines that activate kinases in insulin sensitive organs, impairing the insulin receptor function and interfering with downstream signaling. A consequence of impaired insulin action is the excess of lipid deposition in the liver and adipocytes, elevating lipid metabolites (Hooper & Hooper 2009).

It is now clear that obesity is associated with a state of chronic low inflammation (Wellen & Hotamisligil 2005). The first molecular link between inflammation and obesity, TNF-alpha, was identified when this inflammatory cytokine was discovered to be over-expressed in adipose tissues of rodent models of obesity (Hotamisligil et al. 1993; Sethi & Hotamisligil 1999). As is the case in mice, TNF-alpha is overproduced in the adipose as well as muscle tissues of obese humans (Hotamisligil et al. 1995; Kern et al. 1995; Sazhizadeh et al. 1996).

Pentoxifylline (PTX) is a methylxanthine phosphodiesterase inhibitor with anti-inflammatory effects and immune-regulatory properties (Shan et al. 2012). Early evidence showed that pentoxifylline is able to suppress the synthesis of TNF-alpha *in vitro* and *in vivo,* and to protect animals against endotoxin shock (Zabel et al. 1993). There is now substantial evidence linking TNF-alpha to insulin resistance in humans, animals and *in vitro* systems. Reports (Hotamisligil 1999) suggest that the deletion of TNF-alpha leads to increased insulin sensitivity, i.e., decreased insulin resistance. Furthermore, insulin resistance is an important component of the metabolic syndrome associated with obesity. Early-stage insulin-resistance and related mild glucose intolerance may be compensated by increased insulin secretion (Borst 2004).

The activation of innate immunity, with the subsequent development of a low-grade inflammation, is recognized as a critical factor in the pathogenesis of diabetes mellitus and its complications, including nephropathy. In this sense, there is now evidence for the relevant contribution of pro-inflammatory cytokines, as TNF-alpha, to insulin resistance (Navarro-González et al. 2009). PTX, due to its anti-inflammatory and anti-TNF-alpha properties, may be beneficial in the management of proteinuria/microalbuminuria in diabetic patients (Rodriguez-Morán & Guerrero-Romero 2008; McCormick et al. 2008). Besides possessing anti-inflammatory properties, in part due to its capacity for decreasing TNF-alpha, PTX also reduces lipid peroxidation in diabetic patients (Radfar et al. 2005).

Thus, the objectives of the present study were to evaluate the effects of PTX in alloxan-induced diabetic rats and its mechanisms of action. The focus was on measurements of glycemia, triglyceride levels and other blood biochemical parameters, as well as glycated hemoglobin and fructosamine. Besides, histological and immunohistochemistry analyses for TNF-alpha, iNOS and COX-2 in pancreas, liver and kidney from untreated and PTX-treated diabetic rats were also performed.

## Material and methods

### Drugs and reagents

Alloxan monohydrate was purchased from Sigma Chemical Co. (St. Louis, MO, USA). Pentoxifylline, glibenclamide and metformin were from EMS Laboratory (São Paulo, Brazil) and dissolved in distilled water before use. Kits for blood biochemistry were from Lab Test Diagnóstica SA (Minas Gerais, Brazil). All other reagents were of analytical grade.

### Animals

Male Wistar rats (180–250 g) from the Animal House of the Faculty of Medicine Estácio of Juazeiro do Norte (Brazil) were housed under standard conditions, with proper commercial diet and water *ad libitum.* The study was approved by the Ethical Committee for Animal Research of the Faculty of Medicine of the Federal University of Ceará (No. 01/2011). The experiments were performed according to the Guide for the Care and Use of Laboratory Animals (USA, 1985).

### Experimental protocols

In the 1st protocol, rats were distributed into groups of 10 animals. After 48 h of the alloxan-induced diabetes, blood was collected for measurements of glycemia, triglycerides (TG), total cholesterol and liver transaminases. The animals presenting glycemia levels equal to or higher than 250 mg/dL were submitted to the daily oral administration of PTX (25, 50 or 100 mg/kg), glibenclamide (GLI, 5 mg/kg) or metformin (MET, 50 mg/kg), for 1 week. Normal and untreated diabetic groups received distilled water and were added as negative and positive controls, respectively. In combination experiments, diabetic groups treated with PTX5 + GLI2 or PTX5 + MET5 were also included. One hour after the last drug administration, the animals were weighted and blood collected again for new biochemical measurements. In the 2nd protocol, the same procedure was used, except that the diabetic groups were orally treated with PTX (50 mg/kg), for 1, 2 or 3 months. Water was orally administered to untreated diabetic controls, for the same period.

### Determination of biochemical parameters in sera from alloxan-induced diabetic rats

Blood from the retro-orbital plexus was collected and centrifuged at 3,000 rpm, 10 min, and biochemical parameters (glucose, TG, cholesterol, AST and ALT) were determined by standard procedures, according to the manufacturers’ instructions (Labtest, Brazil).

### Evaluation of PTX hypoglycemic activity on diazoxide-induced hyperglycemia in rats

Groups of 6 to 10 animals were divided into: 1) non-diabetic controls untreated or treated with PTX (50 and 100 mg/kg) or GLI (5 mg/kg) and 2) diabetic rats untreated or treated with PTX (50 and 100 mg/kg) or GLI (5 mg/kg). One hour after treatments and immediately before the diazoxide injection (DZD, 250 mg/kg, i.p.), blood was collected and again at 1, 2 and 3 h after DZD, for glucose determinations.

### Effects of PTX on glycated hemoglobin and fructosamine in diabetic rats

The rats were divided into groups of 6–14 animals. After 48 h of alloxan administration, blood was collected for determination of biochemical parameters. Diabetic animals were daily treated with PTX (5 or 50 mg/kg) or GLI (2 mg/kg) and the association of PTX5 + GLI2, for 1 month (fructosamine) or 2 months (glycated hemoglobin). Untreated diabetic rats (positive controls) were also included for comparisons. Blood measurements were performed again, 1 h after the last administration.

### Histological analyses of untreated and PTX-treated diabetic rats

After decapitation of 3 animals from each group, pancreas, liver and kidney were excised from diabetic rats, untreated or treated for 1 week with PTX (5 and 50 mg/kg), GLI (2 mg/kg) or the association of PTX5 + GLI2. Then, tissue slices were fixed in 10% buffered formalin. Normal tissues were included for comparisons. Paraffin blocks were prepared for routine microscopic slices processing (5 μ sections) and HE staining.

### Immunohistochemistry assays for COX-2, iNOS and TNF-alpha in untreated and PTX-treated diabetic rats

Paraffin sections from pancreas, liver and kidney of untreated (diabetic controls) and PTX-treated (5 and 50 mg/kg, 1 week) diabetic rats were deparaffinized, dehydrated and immersed in 0.1 M citrate buffer (pH 6) under microwave heating (18 min), for antigen recovery. After cooling (20 min), the sections were washed in PBS, followed by endogenous peroxidase blockade. Then, they were incubated overnight (at 4°C) with anti-COX-2 p65, anti-iNOS or anti-TNF-alpha primary antibodies diluted in PBS-BSA, according to the manufacturers’ instructions. At the next day, they were washed in PBS and incubated (30 min) with the biotinylated rabbit secondary antibody. After another washing, the sections were incubated (30 min) with the conjugated streptavidin-peroxidase complex (ABC Vectastain® complex, USA) and. after a final washing, stained with DAB, counter-stained with Mayer hematoxylin, dehydrated and mounted for analyses.

### Statistical analyses

The paired Student’s t test was used in analyses of biochemical parameters, for comparing means of each diabetic group before and after the PTX treatment. In other experiments, One-way ANOVA and the Newman-Keuls as the *post hoc* test were used for comparing results among treatments. The data were considered statistically significant at p < 0.05.

## Results

### Effects of PTX on blood biochemical parameters after 1-week and longer treatments

**Figure 1:**
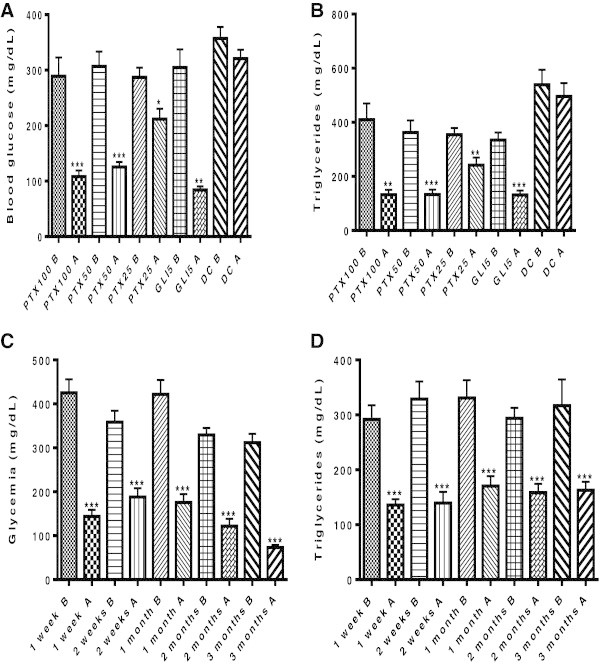
**Effects of pentoxifylline (PTX) treatments (100, 50 and 25 mg/kg, p.o.) on glycemia and triglyceride (TG) levels in diabetic rats. Glibenclamide (GLI, 5 mg/kg, p.o.) was used as reference.** The values are means ± SEM from 6–18 animals per group. **A** and **B**, after 1 week treatment, and **C** and **D** after longer treatments (1 week to 3-months) with PTX (50 mg/kg). **A** (Glycemia), PTX100: ***p < 0.0002; PTX50: ***p < 0.0001; PTX25: *p < 0.0102; GLI5: **p < 0.0014. **B** (TG), PTX100: **p < 0.0013; PTX50: ***p < 0.0003; PTX25: **p < 0.0082; GLI5: ***p < 0.0004. **C** (Glycemia) - PTX50, 1 week: ***q = 42.27; 2 weeks: ***q = 18.78; 1 month: ***q = 31.82; 2 months: ***q = 26.31’; 3 months: ***q = 29.63. **D** (TG) - PTX50, 1 week: ***q = 13.42; 2 weeks: ***q = 18.18;1 month: ***q = 14.75; 2 months: ***q = 12.44; 3 months: ***q = 20.23. Data analyzed by One-way ANOVA, followed by Newman-Keuls as the *post hoc* test or paired Student t test. All comparisons were done after **(A)** vs. before **(B)** treatments, in each group. DC = untreated diabetic controls.

The 1-week treatment of diabetic rats with PTX (25, 50 and 100 mg/kg) decreased from 26 to 63% blood glucose, as related to the same group before treatments. While the treatment of diabetic rats with GLI5 brought glycemia to normal levels, hyperglycemia was maintained in untreated diabetic controls [Figure 1A: F (9, 92) = 24.25, p < 0.0001]. A similar profile was observed in triglyceride levels [Figure 1B: F (9, 86) = 15.20, p < 0.0001]. Total cholesterol and liver transaminases were unaltered. Furthermore, PTX (50 mg/kg) decreased from 60 to 76% glucose levels (mg/dL), after 1-week and longer treatments, as compared to each group before treatments or to untreated diabetic rats, respectively [Figure 1C: F (9, 85) = 291.8, p < 0.0001]. Similarly, triglyceride levels decreased by 50%, along treatments, as related to the same group before treatments [Figure 1D: F (9, 73) = 73.88, p < 0.0001]. Cholesterol and liver transaminases remained unaltered (data not shown).

### PTX hypoglycemic effects were potentiated by glibenclamide but not by metformin

**Figure 2:**
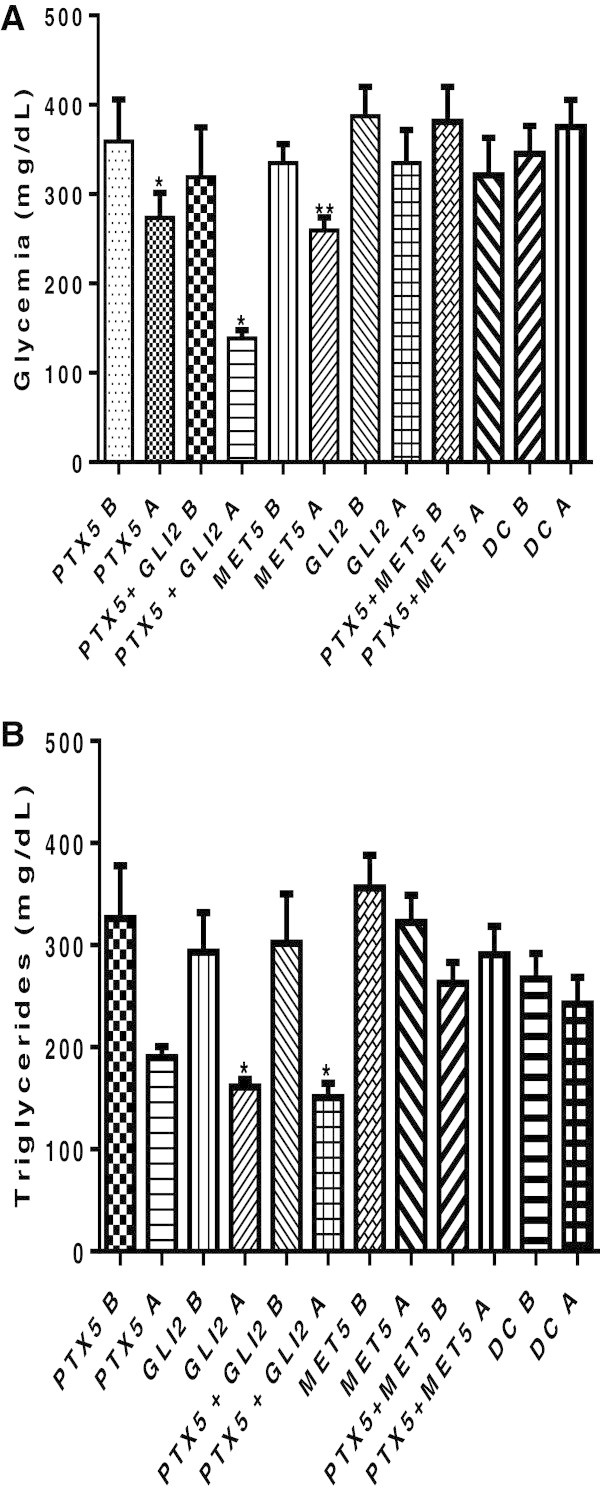
**Effects of a low dose (5 mg/kg, p.o.) of pentoxifylline (PTX), alone or combined to glibenclamide (GLI, 2 mg/kg, p.o.) or metformin (MET, 5 mg/kg, p.o.), on glycemia (A) and TG (B) levels, after 1-week treatment of diabetic rats.** The values are means ± SEM from 5–15 animals per group. PTX5A vs. PTX5B: *p < 0.0443; PTX5A + GLI2A vs. PTX5A: *p < 0.0322; MET5A vs. MET5B: **p < 0.0026. Data analyzed by the paired Student t test. All comparisons were done after **(A)** vs. before **(B)** treatments, in each group.

In order to study the mechanisms of PTX hypoglycemic and hypotriglyceridemic actions, glucose and TG levels were measured in diabetic animals treated with lower doses of PTX (5 mg/kg) or GLI (2 mg/kg) or MET (5 mg/kg), alone or associated, after 1-week treatments. We showed that, while PTX decreased glycemia by 24%, its association with GLI decreased these values by 57%, as related to the same group before treatments or to untreated diabetic groups. Besides, at the 3rd month of treatment, blood glucose levels in this association group were completely normalized (data not shown). On the other hand, in the association of PTX with MET there was no significant decrease in glycemia levels, and MET even blocked the hypoglycemic effect of PTX [Figure 2A: F (11, 90) = 3.603, p < 0.0003]. A similar profile was observed for TG [Figure 2B: F (11, 75) = 4.531, p < 0.0001].

### PTX effects on diazoxide-induced hyperglycemia in non-diabetic and diabetic rats

**Figure 3:**
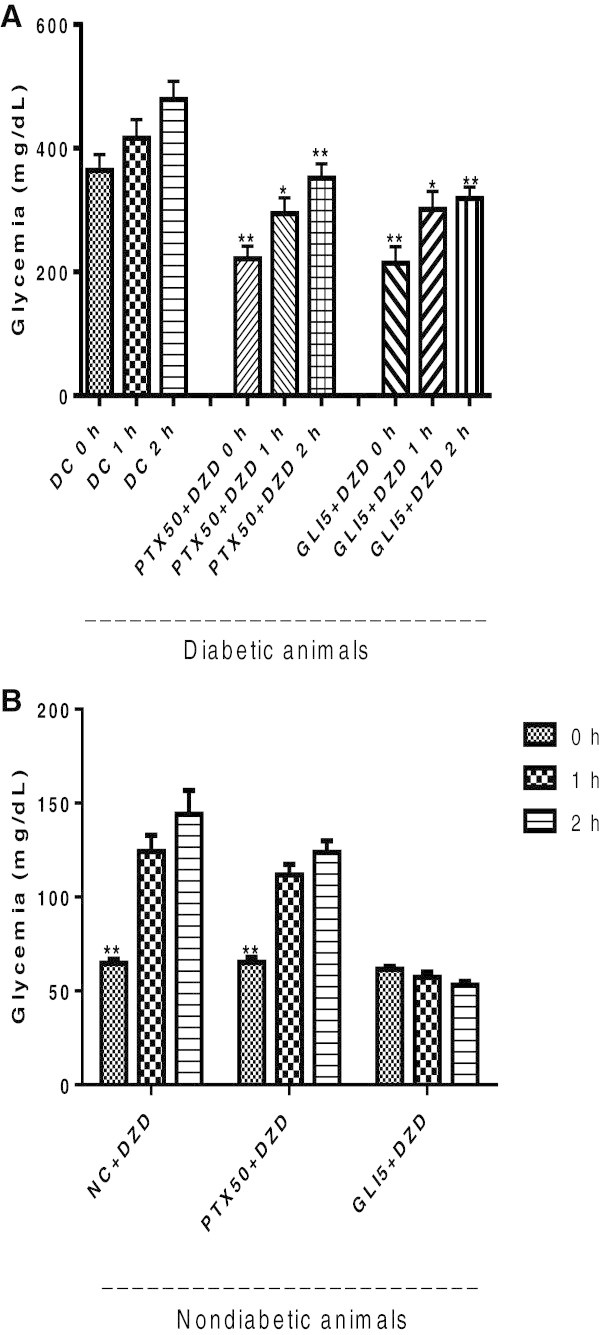
**Effects of pentoxifylline (PTX) on DZD-induced hypoglycemia, in diabetic (A) and non-diabetic (B) rats.** The values are means ± SEM from 6 animals per group. The animals were administered with DZD (250 mg/kg, i.p.). Blood was collected immediately after DZD administration (0 h) and at 1 and 2 h later. DC = untreated diabetic control; DZD = diazoxide. **A** - DC vs. PTX50 + DZD, 0 h: **q = 5.778; 1 h: *q = 4.943; 2 h: **q = 5.130; DC vs. GLI5 + DZD, 0 h: **q = 5.568; 1 h: *q = 4.267; 2 h: **q = 5.941 (One-way ANOVA, followed by Newman-Keuls as the *post-hoc* test). **B** - **p < 0.05 vs. the same group, at 1 and 2 h after DZD administration (paired Student t test).

The results in diabetic rats untreated or treated with PTX or GLI and administered with DZD showed a similar profile among groups, with increases in glycemia values ranging from 1.3 to 1.6 fold, as related to the zero time for each group [Figure 3A: F (8, 45) = 11.12, p < 0.0001]. However, significant and time-dependent increases of 1.9, 2.2 and 2.3 folds were observed for glycemia values in non-diabetic rats untreated or treated with PTX (50 mg/kg), after 1, 2 and 3 h of DZD administration, respectively. On the other hand, no changes were demonstrated for non-diabetic rats treated with GLI (5 mg/kg), after DZD, in the same observation period (Figure 3B).

### PTX decreases fructosamine and glycated hemoglobin in diabetic rats

**Figure 4:**
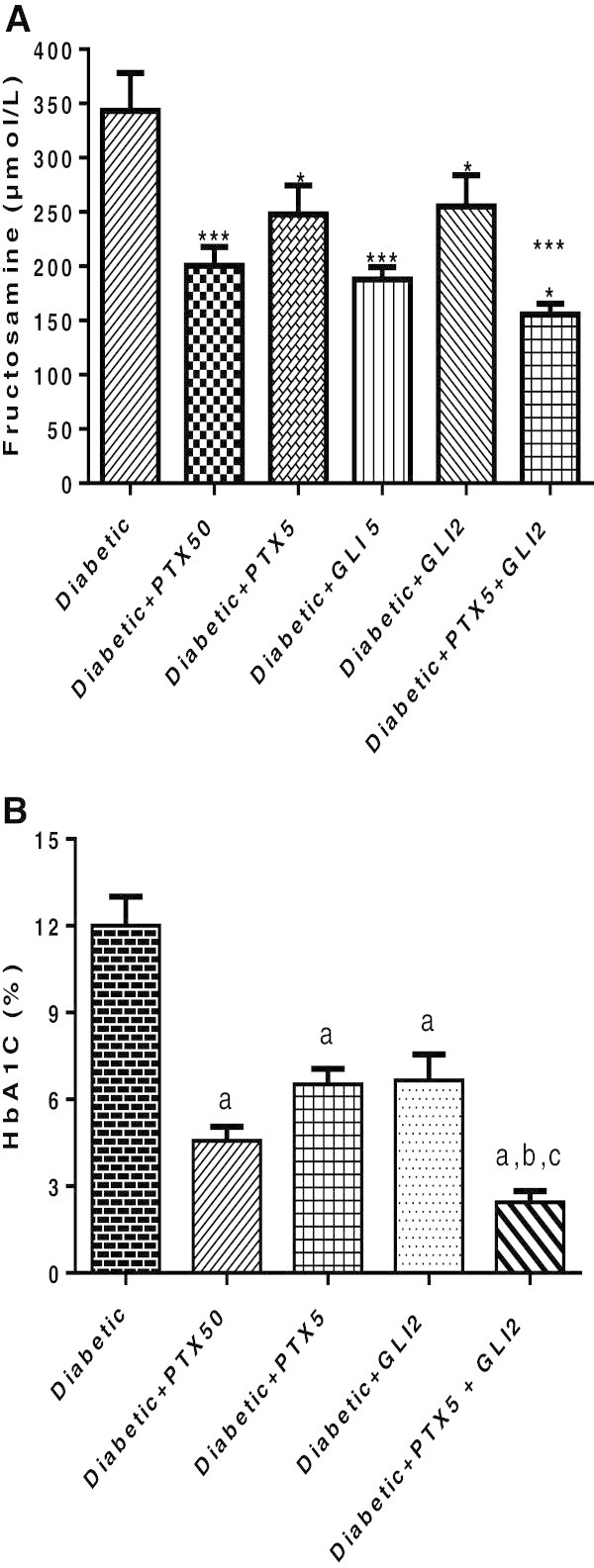
**Effects of pentoxifylline (PTX) on fructosamine levels (A) and glycated hemoglobin (B) in diabetic rats, after 1- or 2-month treatments.** The values are means ± SEM from 6 to 14 animals per group. **A** - DC vs. GLI5: ***q = 6.493; vs. PTX50: ***q = 6.374; vs. PTX5: *q = 3.984; vs. GLI2: *q = 3.827; vs. PTX5 + GLI2: ***q = 8.148; vs. GLI5: ***q = 6.493; PTX5 + GLI2 vs. PTX5: *q = 4.013. **B** - DC vs. PTX50: ***q = 9.118; vs. PTX5: ***q = 7.677; vs. GLI2: ***q = 7.062; vs. PTX5 + GLI2: ***q = 12.32; PTX5 + GLI2 vs. GLI2: ***q = 6.265; vs. PTX5: ***q = 6.505 (One-way ANOVA, followed by Newman-Keuls as the *post hoc* test). DC = untreated diabetic controls; GLI = glibenclamide.

PTX (50 mg/kg) decreased by 42% the fructosamine values in diabetic rats, after 1-month treatment, as related to untreated diabetic rats. At lower doses, PTX (2 mg/kg) or GLI (2 mg/kg) decreased by only 13 and 26% fructosamine values, respectively, while the association of these two drugs caused a 55% decrease, as related to untreated diabetic rats [Figure 4A: F (5, 34) = 8.136, p < 0.0001]. Significant decreases of 62 and 46% were also observed in glycated hemoglobin, 2 months after the alloxan-induced diabetes, in rats treated with PTX (50 or 5 mg/kg), respectively, as related to the untreated diabetic group. While GLI2 alone decreased by 45% glycated hemoglobin, this value decreased by 80%, after the association of PTX5 with GLI2, indicating a potentiation of the effect [Figure 4B: F (4, 41) = 20.45, p < 0.0001].

### PTX reduced histological alterations in pancreas, liver and kidney from diabetic rats

**Figure 5:**
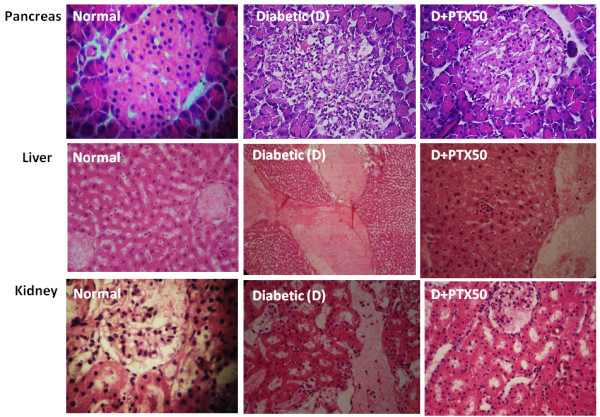
**Representative photomicrographs from HE staining of rat pancreas (upper panel), liver (middle panel) and kidney (lower panel) in normal, untreated diabetic and diabetic rats treated with PTX (5 or 50 mg/kg, p.o.), GLI (2 mg/kg) or the association of PTX5 + GLI2, for 1 week (400X magnification).**

Figure 5 shows representative photomicrographs of histological analyses of pancreas, liver and kidney from untreated and PTX5 and 50- or GLI2-treated diabetic rats, as well diabetic rats treated with the association of PTX5 + GLI2. Normal organs were included for comparisons. Pancreas - untreated diabetic rats: damaged islets with vacuolated cells, pyknotic nuclei, presence of mononuclear inflammatory cells and dilated and ectasic blood vessels; PTX50-treated: almost complete restoration of Langerhans islets; PTX5-treated: moderate damage with vacuolated cells and dilated and ectasic blood vessels; GLI2-treated: almost complete restoration of Langerhans islets; PTX5 + GLI2-treated: diminished damage as related to PTX5-treated rats, suggesting a synergic effect (Figure 5, upper panel). Liver - untreated diabetic rats: hepatocyte cords with dilation of the centerlobular vein; PTX50-treated: hepatocytes with cellular tumefaction and the presence of inflammatory foci (Figure 5, middle panel). Kidney - untreated diabetic rats: presence of tubular epithelium vacuolization and mononuclear inflammatory cells in the inter-tubular space; PTX50-treated: hypocellularity of glomeruli with discrete tumefaction of the tubular epithelium (Figure 5, lower panel).

### Immunohistochemical assays for COX-2, iNOS and TNF-alpha

**Figure 6:**
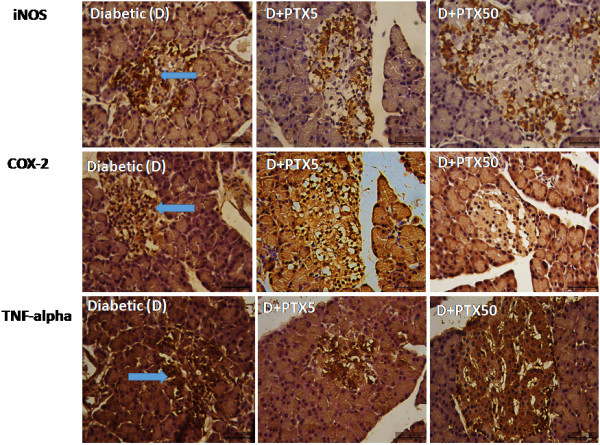
**Representative photomicrographs from immunohistochemistry assays for iNOS, COX-2 and TNF-alpha in pancreas (upper panel), liver (middle panel) and kidney (lower panel) in normal, untreated diabetic (arrows showing immunopositive cells) and PTX-treated (5 and 50 mg/kg) diabetic animals, after 1 week (400X magnification).**

Figure 6 shows representative immunohistochemistry photomicrographs for COX-2, iNOS and TNF-alpha, in pancreas, liver and kidney from untreated (diabetic controls) and PTX-treated (5 and 50 mg/kg, 1 week) diabetic rats. One can observe that, in the three assays, tissues from untreated diabetic rats present a higher immunoreactivity (shown by arrows), as related to those of PTX-treated diabetic rats.

## Discussion

In the present work, we demonstrated that PTX significantly reduces glycemia and triglyceride levels in alloxan-induced diabetic rats, after its daily administration for 1 week. Prolonged treatments bring blood glucose concentrations to normality, while triglycerides decreased by 50%. Our study showed a potentiation of the hypoglycemic effect of PTX by glibenclamide (GLI), after the two drugs association at lower doses. GLI works by inhibiting the ATP-sensitive potassium channels (K_ATP_), in pancreatic beta-cells. This inhibition causes cell membrane depolarization and opening of the voltage-dependent calcium channel, which results in increased intracellular calcium concentration in the beta-cell and subsequent stimulation of insulin release (Luzi & Pozza 1997). PTX, at least in part, shares with GLI this mechanism of action.

Diazoxide (DZD) is used to inhibit inappropriate insulin secretion, causing hypoglycemia (Marks & Samols 1968). Its major mode of action is the opening of K_ATP_ channels in the beta-cell membrane, with repolarization, closure of voltage-dependent Ca^2+^ channels, and lowering of [Ca^2+^] ic (Henquin & Meissner 1982; Trube et al. 1986; Gilon & Henquin 1992). However, DZD has been proposed to decrease the efficacy of Ca^2+^ on exocytosis (Flatt et al. 1994; Renström & Rorsman 1996). In order to investigate whether PTX would block DZD hyperglycemia, as GLI does, the effects of both drugs were studied on diabetic and non-diabetic rats administered with DZD.

Our results show that, in diabetic animals, PTX and GLI presented a similar profile and did not prevent DZD-induced increases in glucose levels. However, in non-diabetic animals, while GLI blocked the increase in glycemia levels after DZD, this effect was not observed after PTX, when the animals treated with PTX + DZD behaved similarly to those treated with DZD only. Such results suggest that other factors, besides the K_ATP_-dependent channels blockade, interfere with the PTX action.

The administration of PTX, for 1 and 2 months, to diabetic rats significantly decreased fructosamine and HbA_1c_ levels, as related to untreated diabetic controls. Both fructosamine and glycated hemoglobin are associated with microvascular conditions in diabetic patients, and increased risk for mortality and morbidity in hemodialysis patients (Selvin et al. 2011; Shafi et al. 2013).

Our histological studies showed that changes in diabetic pancreas were partly reversed after PTX treatment. Interestingly, the diabetic group treated with the association of lower doses of PTX and GLI showed a pancreas with diminished damage, as related to that of diabetic rats treated with PTX alone. Similar results were also observed in diabetic liver and kidney. Previously (MartonJ et al. 1998), a beneficial effect of PTX treatment was demonstrated in rats submitted to acute pancreatitis and showed that PTX very effectively decreased TNF-alpha and IL-6 production under this condition.

Evidences (Coelho et al. 2012) indicate that the administration of PTX, after the onset of acute pancreatitis, decreased levels of pro-inflammatory cytokines.Other studies have shown that PTX exerts a protective effect in animal models of diabetes (Stosic-Grujicic et al. 2001a; Stosic-Grujicic et al. 2001b). These authors showed that the administration of PTX to diabetic animals reduced the production of inflammatory mediators and prevented the development of hyperglycemia. They concluded that these effects of PTX involve down-regulation of the pro-inflammatory cytokine-mediated nitric oxide (NO) synthase pathway. Furthermore, inducible nitric oxide synthase (iNOS) was shown to play a role in fasting hyperglycemia, contributing to hepatic insulin resistance, in a model of obese diabetic mice (Fujimoto et al. 2005). These findings are very similar to ours, except that we used another model and lower PTX doses. Despite these differences, we demonstrated that PTX decreased not only hyperglycemia but also TG levels, in alloxan-induced diabetic rats.

In the present work, we demonstrated that the number of iNOS immune-stained cells was lower in pancreas of diabetic rats, after the PTX treatment. It is largely accepted that the induction of iNOS in pancreatic islets leads to increased NO production associated with dysfunctional beta-cells. Furthermore, a recent study (Muhammed et al. 2012) with diabetic islets culture, exposed to phosphodiesterase (PDE) inhibitors, showed a marked suppression of iNOS mRNA, reduced nitrite production and increased insulin secretion. Considering inflammatory cytokines and NO as potential mediators of pancreatic beta-cell destruction in diabetes, and since PTX is also a PDE inhibitor, these data point out to the same direction as ours and emphasize the potential benefit of PTX in this pathologic condition.

We also demonstrated that PTX treatment decreases the number of cyclooxygenase (COX-2) immunopositive cells in diabetic pancreas. COX-2 has been previously reported (Persaud et al. 2004) to be the dominant isoform and insulin-secretor in beta-cells under basal conditions. The observation that hyperglycemia increases the production of IL-1beta in human beta-cells and induces COX-2 expression, led these authors to suggest this to be a route by which hyperglycemia contributes to beta-cell dysfunction. Another work (Ling et al. 2005) demonstrated that the exposure of pancreatic beta-cells to IL-1beta induces the expression of iNOS and COX-2, and the subsequent formation of NO and prostaglandin E2 (PGE2) may impair beta-cell function. Therefore, the authors concluded that NO-affected COX-2 activity is directly linked to COX-2 gene transcription and protein expression in pancreatic beta-cells, providing a new therapeutic strategy for the management of diabetes mellitus.

In addition, PTX also decreases TNF-alpha immunoreactivity in diabetic pancreas, liver and kidney. Increased TNF-alpha production has been observed in adipose tissue derived from obese rodents or humans, and has been implicated as a causative factor in obesity-associated insulin resistance and diabetes mellitus pathogenesis. Furthermore, current evidence suggests that the administration of exogenous TNF-alpha to animals can induce insulin resistance, whereas its neutralization can improve insulin sensitivity (Moller 2000). Since plasma TNF-alpha is associated to insulin resistance, one can assume that this cytokine plays a significant role in the pathogenesis of chronic insulin resistance in humans (Plomgaard et al. 2007). In addition, PTX (50 mg/kg) administered to rats for 8 weeks was shown to inhibit insulin resistance and prevent TNF-alpha elevation, leukocyte infiltration and endothelial pyknosis (El-Bassossy et al. 2011).

A recent work (Francés et al. 2013) studied the contribution of TNF-alpha intracellular pathway and oxidative stress for the development of apoptosis, in the liver of diabetic rats. Interestingly, iNOS inhibition significantly reduced TNF-alpha levels. These data indicate that the regulation of TNF-alpha and oxidative stress in the diabetic state could be of therapeutic relevance for the improvement of complications linked to chronic hyperglycemia. Furthermore, results from most animal studies and randomized controlled trials on diabetic kidney disease consistently demonstrated that short-term use of PTX produces a significant reduction of proteinuria and microalbuminuria (Badri et al. 2011).

In conclusion, we showed that pentoxifylline decreases blood glucose and TG levels in diabetic rats. The pentoxifylline hypoglycemic effects are similar to those of glibenclamide and, at least partly, related to the inhibition of ATP-sensitive K^+^ channels. It also improves histological changes and decreases the immunoreactivity to iNOS, COX-2 and TNF-alpha, in diabetic pancreas, liver and kidney. Thus, pentoxifylline by its anti-inflammatory and antioxidant properties is a potential and alternative drug for the treatment of diabetes mellitus and its related complications.
